# Investigating Prescriptions and Mechanisms of Acupuncture for Chronic Stable Angina Pectoris: An Association Rule Mining and Network Analysis Study

**DOI:** 10.1155/2020/1931839

**Published:** 2020-10-13

**Authors:** Jie Yu, Yongliang Jiang, Mingqi Tu, Binjun Liao, Jianqiao Fang

**Affiliations:** Department of Neurobiology and Acupuncture Research, The Third Clinical Medical College, Zhejiang Chinese Medical University, Key Laboratory of Acupuncture and Neurology of Zhejiang Province, Hangzhou 310053, China

## Abstract

Chronic stable angina pectoris (CSAP) is a worldwide cardiovascular disease that severely affects people's quality of life and causes serious cardiovascular accidents. Although acupuncture had been confirmed as a potential adjunctive treatment for CSAP, the basic rules and mechanisms of acupoints were little understood. We conducted a systematic search of the China Biology Medicine (CBM), VIP database, Wangfang database, China National Knowledge Infrastructure (CNKI), PubMed, Cochrane Library, Embase, and Web of Science to identify eligible clinical controlled trials (CCTs) and randomized controlled trials (RCTs), from their inception to 18th February 2020. The acupoint prescriptions in the treatment of CSAP were extracted and analyzed based on the association rule mining (ARM) and network analysis. In addition, potential mechanisms of acupuncture for treating CSAP were summarized by data mining. A total of 27 eligible trials were included. Analysis of acupoint prescriptions covered 36 conventional acupoints and 1 experience acupoint, distributing in 10 meridians. The top three frequently used acupoints were PC6, LU9, and ST36. The top three frequently used meridians were the pericardial meridian, lung meridian, and heart meridian. The most frequently used acupoint combinations were LU9 combined with PC6. Besides, network analysis indicated that the core acupoints included PC6, BL15, ST40, and RN17. Moreover, potential mechanisms of acupuncture for treating CSAP involved the regulation of autonomic nerve function, the content of matrix metalloproteinase-9 (MMP-9), volume and the equivalent block of coronary artery calcified plaque (CACP), endothelin (ET), and nitric oxide (NO), neutrophil-lymphocyte ratio (NLR), the content of C-reactive protein (CRP), and tumor necrosis factor-*α* (TNF-*α*). In conclusion, our findings concerning acupoint prescriptions and potential mechanisms in the acupuncture treatment of CSAP could provide an optimized acupuncture treatment plan for clinical treatment of CSAP and promote further mechanism research and network research of CSAP.

## 1. Introduction

CSAP is characterized by episodes of reversible myocardial demand and supply mismatch, related to ischemia or hypoxia, which can be induced by exercise, mood swings, or other stresses [1]. CSAP has become a major health challenge, affecting the health of millions of people around the world. Recent studies have shown that in Europe, 20000 to 40000 individuals of the population per million suffer from angina, and the prevalence rate increases gradually with the growth of age, while in China, the prevalence of angina in those >50 years of age to be 10% [2, 3]. Meanwhile, 10% to 30% of patients with coronary disease still suffer from CSAP in contemporary clinical practice [4]. Moreover, in addition to the symptoms of angina pectoris, studies have shown that patients can also be accompanied by depression, anxiety, sleep disorders, and other emotional symptoms, which greatly affect the quality of life of patients [5, 6]. Conventional therapies such as calcium channel blockers, beta-blockers, nitrates, and revascularization can alleviate symptoms to some extent, but they are often limited by unavoidable side effects and risks and cannot alleviate the accompanying symptoms at the same time [7, 8]. Therefore, it is particularly important to explore adjuvant therapy for CSAP.

Acupuncture is increasingly used around the world, especially in pain control, including neuralgia, inflammatory pain, visceral pain, etc. [9–11]. Previous research studies have revealed that acupuncture combined with conventional drugs can improve angina symptoms, quality of life, and mood disorders [12, 13]. The underlying therapeutic mechanisms may be related to the regulation of several proteases, vasoactive substances, and inflammation mediators [14–16]. Acupuncture prescriptions consist of multiple acupoints, so it is important to scientifically investigate the commonly used acupoint combination, classification, and their underlying mechanisms. Recently, data mining has been applied to acupuncture and moxibustion researches. Previous studies have revealed acupoints and acupoint combination groups frequently used in acupuncture and moxibustion therapy for CSAP, but the association rule mining and network analysis studies on acupuncture therapy for CSAP alone are still lacking.

Therefore, this paper aimed to summarize the prescription, using association rule mining, network analysis, and the underlying mechanisms, so as to optimize the therapeutic effect of acupuncture for treating CSAP.

## 2. Methods

### 2.1. Participants

Patients with CSAP were included in this study. The diagnostic criteria for CSAP were based on the classification criteria of the American College of Cardiology and the American Heart Association, the guidelines for diagnosis and treatment of chronic stable angina pectoris in China, and the nomenclature and criteria for the diagnosis of ischemic heart disease [17–19]. There were no restrictions on age, sex, or race of patients. Patients that combined with other severe diseases were excluded.

### 2.2. Inclusion Criteria for Studies

This study included randomized control trials (RCTs) and clinical control trials (CCTs) of acupuncture therapy for CSAP. Case reports, cross-sectional studies, comments, cohort studies, animal experiments, and reviews were excluded. Only the sole use of acupuncture or acupuncture combined with basic western medicine which was used as a control group was included, and the combined use of other traditional Chinese medicine therapies, such as acupoint application, moxibustion, cupping, etc., was excluded. In addition, comparisons between different acupuncture therapies were also excluded. Eligible included studies should report at least one of the following outcomes, including frequency of angina attacks, the score of visual analog scale (VAS), the Seattle Angina Questionnaire (SAQ), rescue medication intake, the exercise tests, heart rate variability (HRV), ST-segment depression, and the life quality questionnaire.

### 2.3. Search Strategy

We retrieved eight electronic databases from their inception to February 18th, 2020, which included four Chinese databases: China Biology Medicine disc (CBM), VIP database, Wangfang database, China National Knowledge Infrastructure (CNKI), and four English databases: Pubmed, Cochrane Library, Embase, and Web of Science. The search strategies used in PubMed and other English databases were as follows: (“angina” or “angina pectoris” or “stable angina pectoris” or “chronic stable angina pectoris”) and (“acupuncture” or “electro-acupuncture” or “needle” or “needling”). In Chinese databases, Chinese characters with the same meaning were used for literature retrieval. References of the included literature were also screened to supplement the potential eligible trials.

### 2.4. Data Selection and Extraction

Two independent reviewers (Mingqi Tu and Binjun Liao) screened the included databases for related articles from their inception to February 18th, 2020. The eligible articles were imported into NoteExpress 3.2.0 (http://www.inoteexpress.com/aegean/). Moreover, duplicate articles were identified and deleted with NoteExpress 3.2.0. Then, two reviewers reviewed the title, year, author, abstract, and full text independently to determine the inclusion of final eligible studies. Any discrepancies were solved by introducing a third researcher (Jianqiao Fang) for judgment. The following information was extracted from each eligible article: acupoints, meridians, acupuncture prescriptions, and underlying therapeutic mechanisms. The name of acupoints and meridians was based on the nomenclature and location of acupuncture points (2006) [20]. The extracted data from eligible articles were imported into SPSS 19.0 and SPSS modeler 14.1 for further analysis.

### 2.5. Quality Assessment

The Cochrane risk assessment tool was used to evaluate the included studies. The details were as follows: random sequence generation, allocation concealment, blinding of participants and personnel, blinding of outcome assessment, incomplete outcome data, selective reporting, and other bias. Each item was classified as high risk, low risk, and unclear risk. The quality of the included studies was assessed by Jie Yu and Mingqi Tu, respectively. Any discrepancies were solved by introducing a third reviewer (Jianqiao Fang) for judgment.

### 2.6. Data Mining Analysis

First, we summarized the frequency of acupoints and meridians in the treatment of CSAP; both principle acupoints and additional acupoints that were used based on different Chinese syndrome differentiation were extracted for further analysis. Then, we used association rule mining (ARM) to determine the commonly used acupoint group (two or three-acupoint combinations) and network analysis to determine the correlation of different acupoints. Finally, we concluded the underlying mechanism of acupuncture in the treatment of CSAP.

#### 2.6.1. Association Rule Mining

ARM was used as previously described [21–23]. Apriori algorithm in IBM SPSS Modeler 14.1 was used for the ARM analysis. The strength of an association rule could be measured by its support and confidence. We used the support level to determine the probability that *A* and *B* occurred simultaneously. The support of the item set *A*: *s*(*A*) = *σ*(*A*)/*N*; support for rule *A* ⟶ *B*: *s* (*A* ⟶ *B*) = *σ*(*A* ∪ *B*)/*N*. We used confidence to determine how frequently *B* occurred in transactions containing *A*: *c* (*A* ⟶ *B*) = *σ*(*A* ∪ *B*)/*σ*(*A*). The minimum rule confidence level was set as 80%, which indicated when *A* acupoint appeared, the appearance of *B* acupoint would be 80%, and the minimum conditional support was set to 15%. In addition, we used the lift to observe whether the two item sets were dependent on each other, for which value larger than 1 indicated the two item sets depended on each other. Furthermore, we used Heml1.0.3.7 (http://hemi.biocuckoo.org/) to investigate the cooccurrence matrix of acupoints, which was presented as a heat map.

#### 2.6.2. Network Analysis

After data preprocessing and correlation analysis between acupoints, a directed network was constructed to connect acupoints that were used together to summarize prescriptions for CSAP. We converted the text information in the data source to a vector form, marked the annotation name acupoints as the feature vector, and input them to the 0/1 structured acupoints prescription table. Then, the table was imported into IBM SPSS Modeler 14.1 to create association rules flow, which could output the associated node acupoints and their weigh values. Then, we numbered the output nodes, created a list of the combination of nodes list and edges list, which was imported into Gephi 0.9.2 (https://gephi.org/) to perform network image visualization. We chose the “Fruchterman Reingold” algorithm to establish the central aggregation distribution model and the “*k*-core” method to analyze the core acupoints [24, 25].

## 3. Results

### 3.1. Eligible Studies

**Figure 1:**
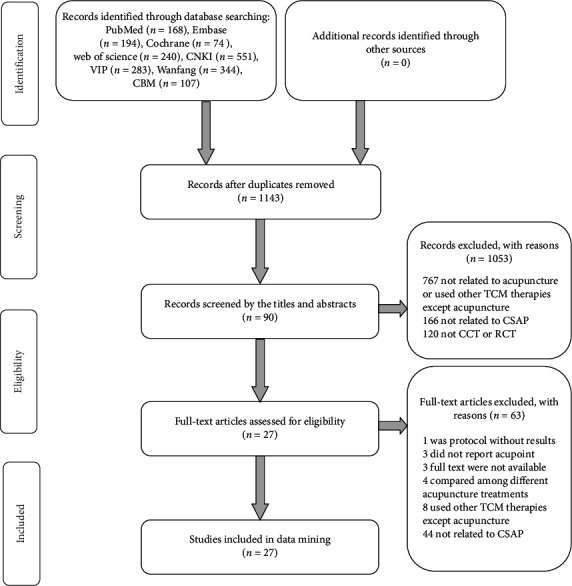
Flow chart of the research process: CBM = Chinese biomedical medicine, CNKI = China national knowledge infrastructure.

The research flow is shown in Figure 1. We identified a total of 1961 related articles and excluded 818 duplicated studies. Then, we screened the remaining 1143 studies through titles and abstracts for 90 potentially eligible studies. After the full text screening, we excluded 63 articles and included 27 studies finally.

### 3.2. Risk of Bias Assessment

**Figure 2:**
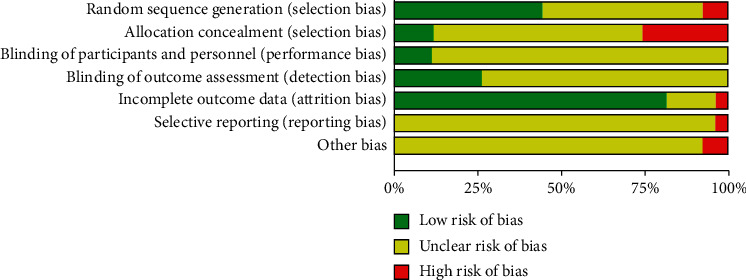
Risk of bias graph.

**Figure 3:**
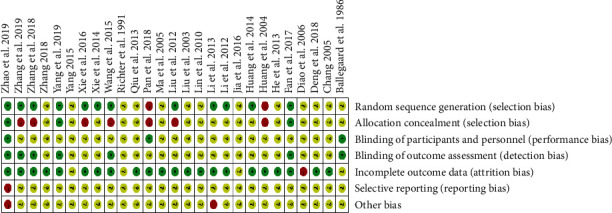
Risk of bias summary.

The risk of bias assessment of the 27 included studies is shown in Table 1. Most studies had an uncertain risk in the domain of allocation concealment, blinding, selective reporting, and other bias. 7 included studies [14, 34, 35, 37, 42–44] used random number table, 3 included studies [16, 46, 48] used a random number, 2 included studies [13, 30] used central randomization, which indicated a low risk in random sequence generation, 1 included study [32] used clinical record number, which indicated a high risk, while other studies had an unclear risk. Most studies had low risk in the domain of incomplete outcome data, which indicated a convincing quality.  Figure 2 demonstrates the risk of bias graph of included trials, and Figure 3 demonstrates a risk of bias summary.

### 3.3. Frequency of Acupoints and Meridians Application for CSAP

A total of 37 acupoints were included, with a total frequency of 130 times (Table 2). Among these acupoints, 36 were traditional acupoints, and one was an experience acupoint that did not belong to any traditional meridian. The top 5 acupoints were PC6, LU9, ST36, BL15, and HT7. A total of 10 meridians were involved in the treatment of CSAP (Table 3), among which pericardium meridian and lung meridian were the most frequently used for 16 times, including 5 and 4 acupoints, respectively. The third most used meridian was the heart meridian, which was used for 11 times and covered 6 acupoints of this meridian. The fourth and fifth most commonly used meridians were the stomach meridian and bladder meridian.

### 3.4. Association Rules of Acupoints for CSAP

**Figure 4:**
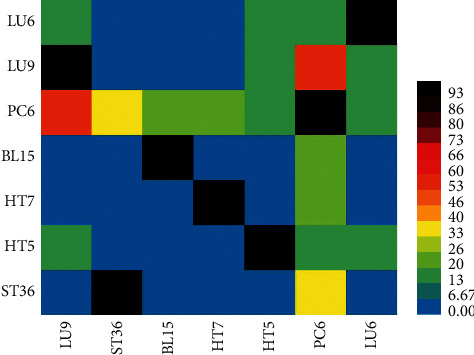
Cooccurrence matrix of acupoints.

The frequency and permutation combination of 36 acupoints in 27 CSAP prescriptions are summarized in Table 4. The top three supports were LU9 with PC6, ST36 with PC6, BL15 with PC6. Among them, LU9 with PC6 had the highest support, with 59.26%. Other association rules included HT7, HT5, LU6 as additional acupoints. The three acupoints combinations included LU6, LU9, and HT5, LU6, PC6, and HT5, LU9, LU6, and PC6, LU9, HT5, and PC6. All the acupoints in different combinations above were dependent on each other, except ST36 and PC6. These results were consistent with the results in the cooccurrence matrix of acupoints (Figure 4). The top paired acupoints were LU9 and PC6, with cooccurrence frequency above 50%.

### 3.5. Network of Acupoints for CSAP

**Figure 5:**
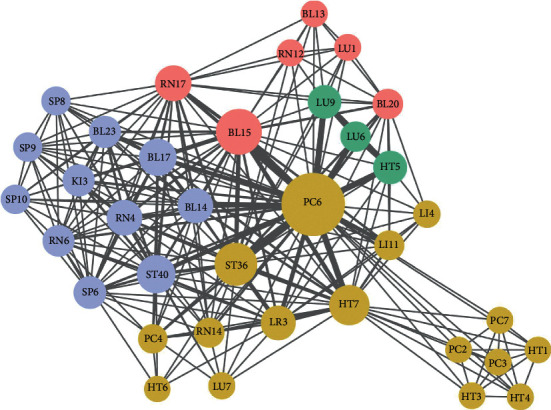
Acupoints association network of acupuncture for CSAP.

**Figure 6:**
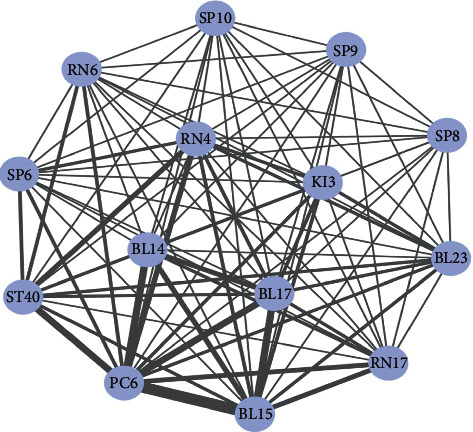
Core acupoints network of acupuncture for CSAP.

After ARM, we finally exported 36 node acupoints and 277 edge weights and constructed a complex network (Figure 5). The average degree of the network model was 23.3, which indicated that each acupoint can be compatible with 23.3 other acupoints on average. There were only four acupoints with a degree greater than 25 (PC6, BL15, ST36, and HT7). Among them PC6 was the most frequently used. The average path length was 1.62 and the network diameter is 2. Furthermore, we used the *K*-core method to explore the core acupoints for CSAP treatment. When *K*-core ≥13, the core acupoints were PC6, BL15, ST40, RN17, etc., which is shown in Figure 6.

### 3.6. Potential Mechanisms of Acupuncture for Treating CSAP

As summarized in Table 5, the underlying mechanisms of acupuncture for treating CSAP included reducing inflammation, stabilizing plaque, dilating blood vessels, increasing blood perfusion, and improving prognosis [14–16, 30, 42, 48]. The role of PC6 and HT5 on the treatment of CSAP included reducing the serum content of matrix metalloproteinase-9 (MMP-9), decreasing content and the equivalent block of coronary artery calcified plaque (CACP) and increasing heart rate variability (HRV) to improve the tension of cardiac autonomic nervous system. Needing at PC6 alone was effective in decreasing endothelin (ET), neutrophil-lymphocyte ratio (NLR), and increasing nitric oxide (NO). The application of multiple acupuncture points could also reduce the content of C-reactive protein (CRP) and tumor necrosis factor-*α* (TNF-*α*).

## 4. Discussion

We used data mining to systematically summarize the prescriptions and mechanisms of acupuncture therapy for CSAP. The top 5 most frequently used acupoints for CSAP in descending order were PC6, LU9, ST36, BL15, and HT7. The most frequently used meridians were pericardium meridian, lung meridian, heart meridian, stomach meridian, and bladder meridian. Among them, the pericardium meridian and heart meridian were indicated to be related to the heart in traditional Chinese medicine [49]. The specificity of meridians and acupoints had been reported previously [50]. The experimental results also showed that compared with nonacupoints and nonmeridians, the acupoints of heart meridian and pericardium meridian could significantly regulate the cardiac function [51, 52]. The sensory afferent of the heart is located in the C8-T5 segment of the spinal cord [53], while the distribution of sensory neurons in the related areas of heart and pericardium meridians is wide. The sensory neurons of the heart meridian and pericardium meridian are relatively concentrated in C7-T1, C6-T2, respectively, which indicated the segmental distribution of the two sensory neurons is roughly the same as that of the heart [54]. In addition, the postsympathetic ganglion cells distributed in the related areas of the heart meridian and pericardial meridian are located in T1-T2 sympathetic ganglion, overlapping with the main part of the postsympathetic ganglion neurons that control the heart, which may be the mechanism of treating heart disease by two meridians [54]. In our study, PC6 was the core acupoint used for CSAP, and the pericardium meridian to which it belongs was also the most frequently used meridian, which is consistent with previous studies. Besides, previous studies had confirmed that the body surface segment of the lung meridian is mainly located in the C6∼T1 segment, which coincides with part of the innervation segment of the heart [27]. Therefore, acupoints along the lung meridian can also be used to treat CSAP. The fourth most frequently used is the stomach meridian, which has a connection with the pericardial meridian in the theory of traditional Chinese medicine [55], so acupoints on the stomach meridian were usually used as auxiliary acupoints for CSAP. In addition, the back-shu points on the bladder meridian, which are located 1.5 inches away from the spine, are particularly important in the treatment of CSAP. There is a close correlation between back-shu points and segmental distribution. The sympathetic neurons that govern the pericardium are located at T1-T5; accordingly, BL14 is located at the level of the fifth thoracic vertebra [56]. Therefore, the back-shu point has the function of regulating the corresponding viscera. These phenomena may indicate that the body surface is related to the viscera.

With regard to acupoint combinations, the top 4 two-acupoint combinations were different combinations of the top 5 acupoints that were most frequently used, which were LU9 plus PC6, ST36 plus PC6, BL15 plus PC6, and HT7 plus PC6. All the acupoints were located on the meridians which had been confirmed to be connected with the heart. However, a three-acupoint combination was seldom used as the prescription in the treatment of CSAP. Therefore, we concluded that acupuncture prescriptions for the treatment of CSAP may be composed of different combinations of commonly used acupuncture points on commonly used meridians, which were pericardium meridian, lung meridian, heart meridian, stomach meridian, and bladder meridian.

In addition, regarding potential therapeutic mechanisms for acupuncture on CSAP, we found that acupuncture could increase HRV, thereby indicating that acupuncture could improve autonomic nerve function. HRV, which indicated the balance between cardiac parasympathetic and sympathetic autonomic influences the heart, had been confirmed to be associated with adverse prognosis in CSAP [57, 58]. Acupuncture can reduce HRV, which also indicates that acupuncture can prevent adverse events of CSAP. Moreover, the effect of acupuncture on CSAP is also related to inflammatory cells and endothelin. Studies had confirmed the relationship between CRP, TNF, and CSAP, which indicated TNF-*α* had an association with left ventricular diastolic dysfunction while CRP was related to the reoccurrence of CSAP [59, 60]. Acupuncture can improve the inflammatory response by reducing these two substances, thereby reducing angina recurrence. NO and ET are two biomarkers in the prediction of coronary complexity. NO can dilate blood vessels while ET can constrict them. Previous researches indicated that ET was related to coronary risks. The dysfunction of NO and ET might cause adverse cardiovascular events [61, 62]. Acupuncture can reduce the content of ET and increase the content of NO, thereby improving blood perfusion. Furthermore, acupuncture also plays a role in stabilizing plaques. Another substance related to CSAP is MMP-9, which had been confirmed to be associated with atherosclerosis, and the increased activity of MMP-9 was found in unstable plaques, suggesting that MMP-9 plays a key role in plaque rupture [63]. Acupuncture can stabilize the plaque by reducing the MMP serum content, which may contribute to reducing coronary adverse events. In summary, acupuncture can not only improve the existing symptoms of CSAP but also improve the prognosis and prevent adverse cardiac events.

## 5. Limitations

It is noteworthy that several limitations of the present study have to be addressed. First, there are few literature studies on acupuncture as adjuvant therapy for angina pectoris, and the quality of the included studies was not high. More high quality, large sample RCTs were needed to improve our results. Secondly, acupoints and meridian types for angina pectoris are single, and the sample size is too small, which limits our analysis of association rules.

## 6. Conclusions

Based on data mining and network analysis, the present study revealed that PC6, LU9, and ST36 were the top 3 acupoints used for CSAP treatment, and the top 3 most used meridians were the pericardium meridian, lung meridian, and heart meridian. The most frequently used acupoint combination was LU9 combined with PC6. And the potential mechanisms of acupuncture in treating CSAP may be related to the regulation of autonomic nerve function, ET, NO, MMP-9, NLR, CRP, and TNF-*α*, which not only improve the existing symptoms but also improve the prognosis and prevent adverse cardiac events.

## Figures and Tables

**Table 1:** Risk of bias of the included studies.

Study	Random sequence generation	Allocation concealment	Blinding		Incomplete outcome data	Selective reporting	Other bias
			Patient/doctor blinding	Outcome assessor blinding			
Ballegaard et al. [26]	Uncertain	Uncertain	Low risk	Low risk	Uncertain	Uncertain	Uncertain
Chang [27]	Uncertain	Uncertain	Uncertain	Uncertain	Low risk	Uncertain	Uncertain
Deng et al. [28]	Uncertain	Uncertain	Uncertain	Uncertain	Low risk	Uncertain	Uncertain
Diao et al. [29]	Uncertain	Uncertain	Uncertain	Uncertain	Low risk	Uncertain	Uncertain
Fan et al. [30]	Central randomization	Low risk	Uncertain	Low risk	Low risk	Uncertain	Uncertain
He et al. [31]	Uncertain	Uncertain	Uncertain	Uncertain	Low risk	Uncertain	Uncertain
Jie et al. [32]	Clinical record number	High risk	Uncertain	Uncertain	Low risk	Uncertain	Uncertain
Huang et al. [14]	Random number table	Uncertain	Uncertain	Uncertain	Low risk	Uncertain	Uncertain
Dan et al. [33]	Uncertain	Uncertain	Uncertain	Uncertain	Uncertain	Uncertain	Uncertain
Li and Li [34]	Random number table	Uncertain	Uncertain	Uncertain	Low risk	Uncertain	Uncertain
Li et al. [35]	Random number table	Uncertain	Uncertain	Uncertain	Low risk	Uncertain	Uncertain
Lin et al. [36]	Uncertain	Uncertain	Uncertain	Uncertain	Low risk	Uncertain	Uncertain
Liu et al. [12]	Uncertain	Uncertain	Uncertain	Uncertain	Low risk	Uncertain	Uncertain
Liu and Zhang [37]	Random number table	High risk	Uncertain	Uncertain	Low risk	Uncertain	Uncertain
Ma et al. [38]	Uncertain	Uncertain	Uncertain	Uncertain	Low risk	Uncertain	Uncertain
Jiang et al. [39]	Clinical record number	High risk	Low risk	Uncertain	Low risk	Uncertain	Uncertain
Youfa and Chen [40]	Uncertain	Uncertain	Uncertain	Uncertain	Low risk	Uncertain	Uncertain
Rlchter Jhaah et al. [41]	Uncertain	Uncertain	Uncertain	Uncertain	Uncertain	Uncertain	Uncertain
Wang et al. [42]	Random number table	High risk	Uncertain	Low risk	Low risk	Uncertain	Uncertain
Shen et al. [43]	Random number	Uncertain	Uncertain	Uncertain	Low risk	Uncertain	Uncertain
Pingchang and Shizhao [44]	Random number table	High risk	Uncertain	Uncertain	Low risk	Uncertain	Uncertain
Yang [45]	Uncertain	Uncertain	Uncertain	Uncertain	Uncertain	Uncertain	Uncertain
Yang et al. [46]	Random number	Low risk	Uncertain	Low risk	Low risk	Uncertain	Uncertain
Zhang [47]	Uncertain	Uncertain	Uncertain	Uncertain	Low risk	Uncertain	Uncertain
Zhang et al. [16]	Random number	High risk	Uncertain	Low risk	Low risk	Uncertain	Uncertain
Zhang et al. [48]	Random number	High risk	Uncertain	Low risk	Low risk	Uncertain	Uncertain
Zhao et al. [13]	Central randomization	Low risk	Low risk	Low risk	Low risk	High risk	High risk

**Table 2:** Frequency of acupoints application for CSAP.

Number	Acupoints	Frequency	Support (%)
1	PC6	25	19.2
2	LU9	16	12.3
3	ST36	9	6.9
4	BL15	7	5.4
5	HT7	6	4.6
6	HT5	5	3.8
7	LU6	5	3.8
8	BL14	4	3.1
9	LI11	4	3.1
10	LR3	4	3.1
11	BL17	4	3.1
12	ST40	4	3.1
13	RN17	4	3.1
14	RN4	3	2.3
15	BL20	2	1.5
16	BL23	2	1.5
17	KI3	2	1.5
18	SP6	2	1.5
19	RN6	2	1.5
20	RN14	2	1.5
21	PC4	2	1.5
22	LI4	1	0.8
23	HT1	1	0.8
24	HT3	1	0.8
25	HT4	1	0.8
26	PC2	1	0.8
27	PC3	1	0.8
28	PC7	1	0.8
29	SP10	1	0.8
30	SP8	1	0.8
31	SP9	1	0.8
32	BL13	1	0.8
33	LU1	1	0.8
34	RN12	1	0.8
35	LU7	1	0.8
36	HT6	1	0.8
37	Xiongtong	1	0.8
Total		130	100.0

**Table 3:** Frequency of meridians application for CSAP.

Meridians	Frequency	Support (%)	Number	Acupoints
Pericardium meridian of hand-jueyin (PC)	16	20.0	5	PC6(25), PC4(2), PC2(1), PC3(1), PC7(1)
Lung meridian of hand-taiyin (LU)	16	20.0	4	LU9(16), LU6(5), LU1(1), LU7(1)
Heart meridian of hand-shaoyin (HT)	11	13.8	6	HT7(6), HT5(5), HT1(1), HT4(1), HT3(1), HT6(1)
Stomach meridian of foot-yangming (ST)	10	12.5	2	ST36(9), ST40(4)
Bladder meridian of foot-taiyang (BL)	8	10.0	6	BL15(7), BL14(4), BL17(4), BL20(2), BL23(2), BL13(1)
Ren meridian (RN)	7	8.8	5	RN17(4), RN4(3), RN6(2), RN14(2), RN12(1)
Large intestine meridian of hand-yangming (LI)	4	5.0	2	LI11(4), LI4(1)
Liver meridian of foot-jueyin (LR)	4	5.0	1	LR3(4)
Spleen meridian of foot-taiyin (SP)	2	2.5	4	SP6(2), SP10(1), SP8(1), SP9(1)
Kidney meridian of foot-shaoyin (KI)	2	2.5	1	KI3(2)

**Table 4:** Association rules of acupoints for CSAP.

Number	Acupoints	Support (%)	Confidence (%)	Lift	Frequency
1	LU9, PC6	59.26	93.75	1.01	16
2	ST36, PC6	33.33	88.89	0.96	9
3	BL15, PC6	25.93	100.00	1.08	7
4	HT7, PC6	22.22	100.00	1.08	6
5	LU6, HT5	18.52	80.00	4.32	5
6	LU9, LU6	18.52	100.00	1.69	5
7	LU6, PC6	18.52	100.00	1.08	5
8	HT5, LU9	18.52	80.00	1.35	5
9	HT5, PC6	18.52	100.00	1.08	5
10	LU6, LU9, HT5	18.52	80.00	4.32	5
11	LU6, PC6, HT5	18.52	80.00	4.32	5
12	LU6, LU9, PC6	18.52	100.00	1.08	5
13	LU9, HT5, PC6	18.52	80.00	1.35	5

**Table 5:** The potential mechanisms of acupuncture on CSAP.

Acupoints	Beneficial effects	Potential mechanisms	Ref. #
PC6, HT5	Delay the process of plaque instability, reduce the incidence of malignant cardiovascular events	Reduce the serum content of matrix metalloproteinase-9(MMP-9)	[15]
PC6, HT5	Stabilize atherosclerotic plaque	Decrease volume and the equivalent block of coronary artery calcified plaque (CACP)	[23]
PC6	Vasodilatation, lowering blood pressure, inhibiting platelet adhesion and aggregation, maintaining normal cardiac output, controlling collateral circulation opening, adjusting tissue perfusion, and protecting myocardial perfusion	Decrease endothelin (ET) and increase nitric oxide (NO)	[16]
PC6, HT5	Improve the tension of the cardiac autonomic nervous system	Increase heart rate variability (HRV)	[24]
PC6	Improve the prognosis of CSAP	Decrease neutrophil-lymphocyte ratio (NLR)	[25]
PC6, RN17, HT6, SP10, LR3	Reduce inflammatory response	Reduce the content of C-reactive protein (CRP) and tumor necrosis factor-*α* (TNF-*α*)	[14]

## Data Availability

The datasets used and/or analyzed during the current study are available from the corresponding author upon reasonable request.

## Abbreviations

ARM: Association rule mining
CACP: Coronary artery calcified plaque
CBM: China biology medicine
CCT: Clinical controlled trial
CNKI: China National Knowledge Infrastructure
CRP: C-reactive protein
CSAP: Chronic stable angina pectoris
ET: Endothelin
NLR: Neutrophil-lymphocyte ratio
NO: Nitric oxide
MMP-9: Matrix metalloproteinase-9
TNF-*α*: Tumor necrosis factor-*α*.
